# Hypersensitivity to fennel is frequent in peach allergic patients and LTP is a major fennel allergen

**DOI:** 10.1186/2045-7022-1-S1-P78

**Published:** 2011-08-12

**Authors:** Chrysi Stafylaraki, Laura Farioli, Joseph Scibilia, Maria Grazia Giuffrida, Ambra Mascheri, Valerio Pravettoni, C Baro, Marta Piantanida, Laura Primavesi, Michele Nichelatti, Alessandro Marocchi, Jan Walter Schroeder, Elide Anna Pastorello

**Affiliations:** 1 Niguarda Cà Granda Hospital, Allergology and Immunology Unit, Milan, Italy; 2 Niguarda Cà Granda Hospital, Department of Laboratory Medicine, Milan, Italy; 3 National Research Council, ISPA, Turin, Italy; 4 Foundation IRCCS Ca’ Granda Ospedale Maggiore Policlinico, Clinical Allergy and Immunology Unit, Milan, Italy; 5 Niguarda Cà Granda Hospital, Center for Clinical Research, Milan, Italy

## Background

fennel is usually consumed as seeds in Northern Europe, while in the Mediterranean area the plant is also consumed fresh. Because of the low consumption there aren't many studies that regard the identification of fennel allergens in the literature.

## Objective

to study 1) the possible correlation between severe allergic symptoms to peach and severe symptoms to fennel; 2) identify fennel allergens; 3) evaluate whether the rPru p 3 recombinant allergen could help identify subjects with severe reactions to fennel.

## Methods

within a population of patients with peach allergic symptoms of variable severity we investigated which patients also had documented allergy to fresh fennel (Clinical Trials.gov, protocol ID NCT00715156). We examined the type of allergic reaction to fennel by means of a clinical questionnaire, skin prick test, prick-prick with fresh fennel, open challenge and IgE-specific levels to fennel and to the following recombinant allergens: anti-rPru p 1, 3, 4, anti-rBet v 1, 2 and 4. We compared the different clinical reactions to fennel between patients with mild symptoms to peach (group A, 21pts) and patients with severe systemic symptoms to peach (group B, 16 pts). SDS-Page and IgE immunoblotting were performed with fennel extract and the N-terminal sequences of the allergenic molecules were determined analyzing the proteins eluted by SDS-gel on a protein sequencer. Immunoblotting inhibition was performed to evaluate the cross-reactivity between fennel and peach extract.

## Results

we found a significant association between severe symptoms to fennel and peach induced severe symptoms (p=0.0081). IgE immunoblotting showed that more than half of the patients reacted toward a IgE-binding protein of about 9 kDa. The aminoacids of the N-terminal sequence were Ala-Ile-Thr-Xxx-Gly-Qln-Val-Thr-Ser-Lys-Leu-Gly, corresponding to a nsLTP. The immunoblotting inhibition experiment with peach extract showed a total inhibition of IgE binding to the 9 kDa band of fennel.

## Conclusion

Peach and fennel allergens are correlated and fennel is a food that should be considered in the LTP syndrome.

